# Fragment-Based Screening Identifies Novel Non-Amino Acid Inhibitors of the Sodium-Coupled Neutral Amino Acid Transporter SNAT2

**DOI:** 10.1007/s11095-025-03902-7

**Published:** 2025-08-08

**Authors:** Sebastian Jakobsen, Carsten Uhd Nielsen

**Affiliations:** Department of Physics, Chemistry and Pharmacy, University of Southern Denmark, Campusvej 55, 5230 Odense M, Denmark

**Keywords:** Amino acid transporter, FMP assay, Fragment-based screening, Inhibitor, SNAT2

## Abstract

**Introduction:**

Amino acid transporters like the sodium-coupled neutral amino acid transporter 2 (SNAT2, SLC38A2) have gained interest for their roles in, e.g., the central nervous system and in cancer. Efforts in discovering inhibitors against these transporters often result in amino acid-based inhibitors that lack selectivity and are likely to compete with amino acid substrates to bind their targets. To circumvent this, we aimed to discover novel non-amino acid inhibitors of SNAT2 by screening a library of fragment-sized compounds.

**Methods:**

320 fragment compounds were screened for their inhibition of ^3^H-Gly uptake in hyperosmotically upregulated SNAT2-expressing PC-3 cells. The top five hits were studied further for their inhibitory potency and structure–activity relationship (SAR). Their ability to be translocated by SNAT2 was studied using the FLIPR membrane potential (FMP) assay, as well as their mechanism of inhibition.

**Results:**

The screen revealed two similar scaffolds that showed SNAT2 inhibition, namely 1,3-benzothiazole-2-amine and 1,3-benzoxazole-2-amine. The SAR revealed how hydrophobic substituents at specific positions were needed for the structures to show SNAT2 inhibition. The best inhibitors inhibited SNAT2 with IC_50_s in the range of 0.64–1.08 mM. Many of the fragment compounds showed an apparent hyperpolarization in the FMP assay, making it difficult to determine their ability to be translocated by SNAT2. An allosteric mechanism of inhibition was implied for the thiazole and oxazole scaffolds, as these resulted in inhibition patterns that resembled non- or un-competitive inhibitors.

**Conclusion:**

In conclusion, we discovered multiple novel non-amino acid compounds that inhibited SNAT2 and can serve as starting points for the further development of SNAT2 inhibitors.

**Supplementary Information:**

The online version contains supplementary material available at 10.1007/s11095-025-03902-7.

## Introduction

Amino acids are important in regulating cell growth and metabolism and act as neurotransmitters or sources of neurotransmitters in the central nervous system (CNS). Amino acid transporters (AATs) have recently gained interest as pharmacological targets primarily for their role in cancer in addition to their more established role as drug targets in the CNS [1, 2]. In cancer, it is primarily the supply of glutamine, which fuels the glutaminolysis pathway, that has been targeted by AAT inhibitors along with the amino acid-induced activation of the cell growth regulator mTORC1 (mammalian target of rapamycin complex 1) [2, 3]. An example of the therapeutic use of targeting AATs is exemplified by JPH203 (nanvuranlat), an inhibitor of the large amino acid transporter LAT1 (SLC7A5), which is currently in clinical trials for the treatment of biliary tract cancer [4]. However, AAT inhibitors are, in most cases, based on an amino acid scaffold, which comes with a number of challenges. Firstly, the similar amino acid core has often proven to give selectivity issues, given that there are more than 60 different AATs to accommodate 20 proteinogenic amino acids [1]. Furthermore, amino acid-based inhibitors are expected to bind to the substrate binding site of the AATs and thus compete with the natural substrates for binding. Therefore, it might be more beneficial to target allosteric sites of AATs. For instance, it has been shown that AATs of the SLC1 subfamily have a conserved allosteric site that has been successfully targeted by inhibitors [5].

An AAT, which is underexplored in terms of inhibitor identification, is the sodium-coupled neutral amino acid transporter SNAT2 (SLC38A2). This ubiquitously expressed AAT is characterized by a substrate preference for small- to medium-sized neutral amino acids like alanine and glutamine and is highly upregulated by amino acid starvation and hyperosmotic stress [6–8]. SNAT2 has been implicated in cancers, such as gastric and breast cancer [9, 10], and might also play a role in the glutamate-glutamine cycle in the brain [11]. SNAT2 inhibition has, at this point, primarily been based on amino acid analogs with IC_50_s in the range of 0.2–2 mM [12], but a single non-amino acid inhibitor named MMTC has been proposed [13]. However, MMTC was not able to show SNAT2 inhibition in a different study using a different cell line [14]. The 3D-structure of SNAT2 has yet to be solved, therefore making *in silico* drug discovery campaigns challenging. This leaves *in vitro* screening approaches as the remaining viable option to discover novel inhibitor scaffolds, but they can be quite costly and time-consuming. Alternatively, fragment-based screening offers a more efficient way to screen for new scaffolds, using small compounds that are often more soluble, where the hits often have moderate affinities, in the millimolar range, that can serve as a molecular starting point for finding new inhibitors. Since chemical space is sampled more efficiently with the low molecular weight fragment compounds compared to high-throughput approaches, a much smaller library is needed, making the screening process less demanding [15]. While these fragment-based methods are relatively new within the field of transporters, they have been used effectively to find inhibitors of SLC25A20 [16], as well as more soluble inhibitors of the ABC transporter BCRP (ABCG2) [17].

Here, we have aimed to uncover non-amino acid inhibitors of SNAT2 by broadly and efficiently searching for novel scaffolds that can serve as starting points for the development of inhibitors. To do so, we screened a diverse library of fragment-sized compounds for their inhibition of SNAT2. Based on the initial screen, we then explored the structure–activity relationship of the hit structures and determined their inhibitory potency. Furthermore, we used the FLIPR membrane potential (FMP) assay to investigate if these novel ligands were translocated by SNAT2 to establish if they were substrates or inhibitors. Following this, we investigated their mechanism of inhibition. Our results present a novel class of non-amino acid SNAT2 inhibitors that do not compete with substrate binding and, thus, seemingly work through an allosteric mechanism.

## Materials and Methods

### Materials

[2-^3^H]-Glycine (45.2 Ci ⋅ mmol^−1^), L-[3,4,5-^3^H(N)]-leucine (72.6 Ci ⋅ mmol^−1^), Ultima GoldTM scintillation fluid, and scintillation vials (6 mL, Pony VialTM) were from Perkin Elmer/Revvity (Waltham, MA, USA). Dulbecco's Modified Eagle Medium/Nutrient Mixture F-12 (DMEM/F12), penicillin/streptomycin (100x), L-glutamine (200 mM), and trypsin–EDTA (10x) were from Dominique Dutscher (Bernolsheim, France). Fetal bovine serum (FBS), sodium pyruvate (100 mM), phosphate-buffered saline (PBS), and sodium bicarbonate solution (7.5%) were from Biowest (Nuaillé, France). Hank’s Balanced Salt Solution (HBSS, Gibco) and XTT sodium salt were from Thermo Fischer Scientific (Waltham, MA, USA). Phenazine methyl sulfate (PMS) (> 98.0%) and O-benzyl-L-serine (≥ 99.0%) were from VWR (Radnor, PA, USA). L-Ascorbic acid, 4-(2-hydroxyethyl)−1-piperazineethanesulfonic acid (HEPES) (≥ 99.5%), D-(+)-raffinose pentahydrate (≥ 98.0%), Triton-X 100, L-glutamine (≥ 99.5%), L-arginine hydrochloride (≥ 98%), glycine (98%), L-leucine (> 99.5%), and sodium dodecyl sulfate (SDS) (≥ 98.5%) were from Sigma Aldrich (Merck KGaA, Darmstadt, Germany). Dimethyl sulfoxide (DMSO) was from AppliChem GmbH (Darmstadt, Germany). JPH203 (99.73%) was from Selleck Chemicals (Houston, TX, USA). The Essential Fragment Library was from Enamine (Kyiv, Ukraine). The compounds **1**–**28** were from either BLDpharm (Shanghai, China) or Enamine (Kyiv, Ukraine) (Supplementary Information, Table S1).

### Cell Culture

PC-3 cells (ECACC 90,112,714) were obtained from the European Collection of Authenticated Cell Cultures (ECACC;UK Health Security Agency, Salisbury, UK) and were received in passage 31. PC-3 cells were kept in DMEM/F12 supplemented with 10% fetal bovine serum (FBS), 100 U ⋅ mL^−1^ penicillin, 0.1 mg ⋅ mL^−1^ streptomycin, 2 mM L-glutamine, 2 mM sodium pyruvate, and 20 μg ⋅ mL^−1^ L-ascorbic acid. The cells were kept in an incubator at 37 °C in an atmosphere of 5% CO_2_ and with 94–97% relative humidity, and the culture medium was changed every 2–3 days. For all studies, the PC-3 cells were seeded in 96-well plates (area 0.32 cm^2^) with a density of 1.5 ⋅ 10^5^ cells ⋅ cm^−2^ two days before the experiment. Clear 96-well plates were used for uptake studies, white plates for CellTiter-Glo viability studies, and black plates for FLIPR membrane potential (FMP) assays. For hyperosmotic stimulation, PC-3 cells were incubated in hyperosmotic media 24 h before the experiment. The hyperosmotic media was prepared by supplementing normal isoosmotic culture medium with 200 mM raffinose to reach an osmolality of approximately 510 mOsm ⋅ kg^−1^ (516 ± 15 mOsm ⋅ kg^−1^). Experiments were performed on PC-3 cells in passages 37–51 (2–16 after thawing).

### Radiolabeled Uptake Studies

^3^H-Gly uptake was studied in hyperosmotically treated PC-3 cells. Solutions were prepared using Hanks Balanced Salt Solution (HBSS), which consisted of the following (mM): CaCl_2_, 1.26; MgCl_2_, 0.49; MgSO_4_, 0.41; KCl, 5.33; KH_2_PO_4_, 0.44; NaCl, 138; Na_2_HPO_4_, 0.34; D-glucose, 5.56; NaHCO_3_, 4.17. HBSS was supplemented with 10 mM HEPES and adjusted to pH 7.40 ± 0.01 (HBSS*) with 0.1–5.0 mM NaOH. Donor solutions contained 0.5 µCi ⋅ mL^−1^ of ^3^H-Gly (11.1 nM). For substrate concentration-dependent studies, the concentration of the substrate was achieved by supplementing with cold Gly. After aspirating the media from the cells, the cells were preincubated in HBSS* for 15 min at 37 °C and 220 rpm. To begin the uptake study, the buffer was removed, and 80 µL of prewarmed (37 °C) donor solution was introduced to the cells, followed by incubation at 37 °C and continuous rotation at 220 rpm using a Talboys incubating microplate shaker (Troemner, Thorofare, NJ, USA). After 5 min (or other uptake time if stated), the uptake process was stopped by removing the donor solutions through vacuum suction, and the cells were rinsed three times with 100 µL of ice-cold HBSS. To detach the cells, 50 µL of 0.1% Triton-X 100 was applied for at least 25 min. The detached cells were then transferred to scintillation vials, along with 2 mL of Ultima Gold scintillation fluid. The vials were vortexed, and disintegrations pr minute (DPM) were measured using a TriCarb 4910TR liquid scintillation counter (PerkinElmer, Waltham, MA, USA).

^3^H-L-Leu uptake was studied similarly, using isoosmotically treated PC-3 cells and donor solutions containing 0.2 µCi ⋅ mL^−1 3^H-L-Leu (2.75 nM) instead.

### Screening of Fragment-Sized Compound Library

The ^3^H-Gly uptake assay was used to screen the Essential Fragment Library, which contains 320 compounds. The fragment library was received as 100 mM DMSO stock solutions and diluted to a final concentration of 0.5 mM using HBSS*. The screening concentration of 0.5 mM was chosen because it is similar to the K_m_ values of the natural substrates of SNAT2 [6]. The library was split into four 96-well plates, taking up 10 columns in each plate, with the remaining two columns being used for buffer controls and positive controls (20 mM L-Gln). Each compound was tested in a single replicate in a single cell passage. Compounds inhibiting ^3^H-Gly uptake by > 35% were considered hits.

### CellTiter-Glo Viability Assay

The CellTiter-Glo® 2.0 Cell Viability Assay (Promega, Madison, WI, USA) was used to assess the possible cytotoxic effects of the tested compounds. 0.5 mL of HBSS* was added to 2.5 mL of the CellTiter-Glo reagent. Hyperosmotically treated PC-3 cells were preincubated in HBSS* for 15 min at 37 °C and 220 rpm. The cells were then treated with 50 µL of the compounds of interest for 10 min at 37 °C and 220 rpm. After removing the compound solutions, 50 µL of room temperature HBSS* was added to each well, followed by 30 µL of the CellTiter-Glo reagent. The plate was then placed in a CLARIOstar® Plus plate reader from BMG LABTECH (Ortenberg, Germany) at room temperature and was then shaken for 2 min at 200 rpm. Luminescence was measured after 5, 10, 15, and 20 min, and the timepoint resulting in the highest signals was used. The luminescent signals were normalized to control wells exposed only to HBSS*.

### FLIPR Membrane Potential (FMP) Assay

The FLIPR membrane potential (FMP) assay was used to assess the ability of compounds to be translocated by SNAT2, which causes a membrane depolarization through the coupling movement of Na^+^ ions as previously described [12]. To briefly describe the method, hyperosmotically treated PC-3 cells were first preincubated in HBSS* for 10 min, followed by 30 min of incubation with 50 µL 1 × FMP probe (0.55 mg ⋅ mL^−1^, Blue component A from Molecular Devices, San Jose, CA, USA) at 37 °C and 220 rpm. The plate was then placed in a CLARIOstar® Plus plate reader from BMG LABTECH (Ortenberg, Germany) at 37 °C to measure the fluorescence (Ex. 530 nm, Em. 565 nm) of each well, one column at a time. First, the baseline was measured for 30 s by measuring each well every 3 s. The plate was then removed from the reader, and 50 µL of working solutions containing 1 × FMP probe and 2 × the final concentration of compounds of interest was added. After the addition of the substrate solution, the plate was returned to the reader and shaken at 200 rpm for 5 s, and then fluorescence intensity was measured in 3-s intervals for 120 s.

### Data Analysis

To evaluate the screening assay, the Z’ factor was calculated, which measures the separation of positive and negative controls relative to their standard deviation [18]. The Z’ factor is defined by **Eq. **(1).

1 $${Z}^{\prime}factor=1-\frac{3\left({\sigma }_{p}+{\sigma }_{n}\right)}{\left|{\mu }_{n}-{\mu }_{p}\right|}$$

where σ_p_ is the SD of the positive control, σ_n_ is the SD of the negative control, μ_p_ is the average of the positive control, and μ_n_ is the average of the negative control.

For concentration-dependent inhibition studies, GrahpPad Prism 10.1.2 was used to fit the data to **Eq. **(2).

2 $$Y=Bottom+\frac{Top-Bottom}{1+{\left(\frac{I{C}_{50}}{X}\right)}^{n}}$$

where *Y* is the % uptake, *X* is the concentration of the inhibitor, *Top* and *Bottom* are the top and bottom plateaus respectively, IC_50_ is the half maximal inhibitory concentration, and n is the Hill slope. Generally, a three-parameter model was used where the Hill slope is constrained to a standard value of −1. In the case of curves that appeared to have a different steepness, a custom Hill slope was fitted (four-parameter model). For the fragment compounds, a full IC_50_ curve was generally not able to be defined within the solubility limits of the compounds, so the Top was constrained to 100% and the Bbottom constrained to 10%, equaling that of the positive control (20 mM L-Gln).

For the FMP assay, the baseline was defined as the mean fluorescence of the first data points before substrate addition. The baseline of each well was then subtracted from the data points to get the ΔF. Plotting ΔF as a function of time and calculating the area under the curve (AUC) after substrate addition was used as a measure of SNAT2 activity.

For substrate concentration-dependent uptake studies, used to determine the mechanism of inhibition, the DPM achieved from the positive control (20 mM L-Gln) was subtracted as the background, before converting DPM to uptake rates. The resulting uptake rates were fitted to the Michaelis–Menten equation (**Eq. **(3)) using GraphPad Prism 10.1.2.

3 $$V=\frac{{V}_{max}\cdot \left[S\right]}{{K}_{m}+\left[S\right]}$$

where V is the uptake rate, V_max_ is the maximum uptake rate, [S] is the substrate concentration, and K_m_ is the Michaelis constant, which reflects the concentration where an uptake rate 50% of V_max_ is achieved.

### Statistical Analysis

Values are reported as means ± standard deviation (SD) calculated from *N* observations across *n* cell passages unless otherwise stated. The standard error (SE) reported by GraphPad Prism is used to show the variability of fitted parameters (e.g., IC_50_ values, K_m_ values). One-way or two-way ANOVAs were performed, followed by either Dunnett's or Šidák’s multiple comparisons test, to test for statistically significant (p < 0.05) differences.

## Results and Discussion

### Screening a Fragment-Based Library

**Fig. 1:**
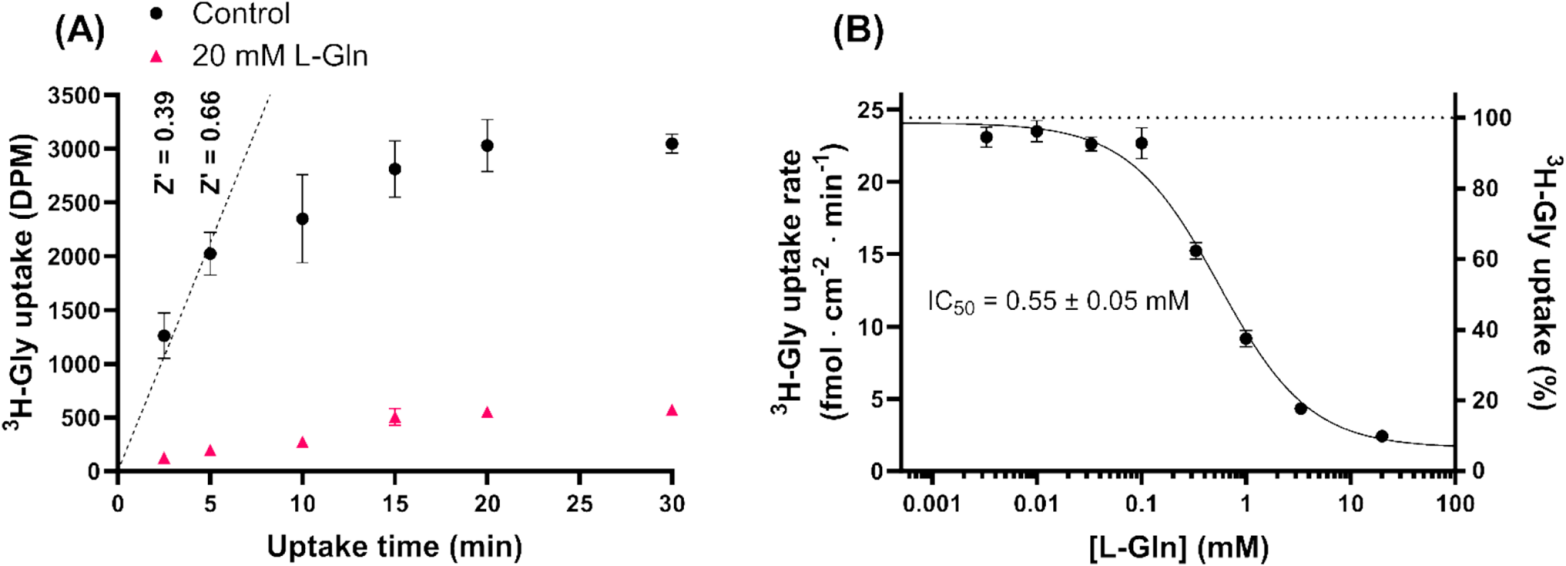
Prescreening study of the ^3^H-Gly uptake assay in hyperosmotically treated PC-3 cells. (**A**) Time-dependent uptake of ^3^H-Gly (11.1 nM) in the presence or absence of 20 mM L-Gln (*n* = 1, *N* = 4). A straight line is fitted to the first two points and forced through zero to indicate linearity. Z’ is calculated using **Eq. **(1). (**B**) Concentration-dependent inhibition of a 5-min ^3^H-Gly (11.1 nM) uptake by L-Gln (*n* = 1, *N* = 3). The points were fitted to **Eq. **(2) to determine the IC_50_ value (± SE). All experiments used HBSS*. The cells were exposed to 0.5 µCi ⋅ mL^−1 3^H-Gly (11.1 nM) at 37 °C. Values are reported as means ± SD

In the search for novel SNAT2 inhibitors based on non-amino acid scaffolds, the Essential Fragment Library was chosen as a compound library for a screen against SNAT2 activity. Before initiating the screen, our previously described ^3^H-Gly uptake assay in hyperosmotically treated PC-3 cells, known to upregulate SNAT2 [14, 19], was adapted to a 96-well format and tested for its suitability as a semi-high-throughput screening assay. The assay showed a linear correlation between uptake and uptake time within the first 5 min (Fig. 1A). A 5-min uptake allowed for a better separation between the control and positive control (20 mM L-Gln, a SNAT2 substrate [6]), which is reflected in the Z’ factor of 0.66, indicating an excellent assay for screening [18]. L-Gln inhibited ^3^H-Gly uptake with an IC_50_ of 0.55 mM (Fig. 1B), further substantiating its use at 20 mM as a positive control for the fragment compound screen.

**Fig. 2:**
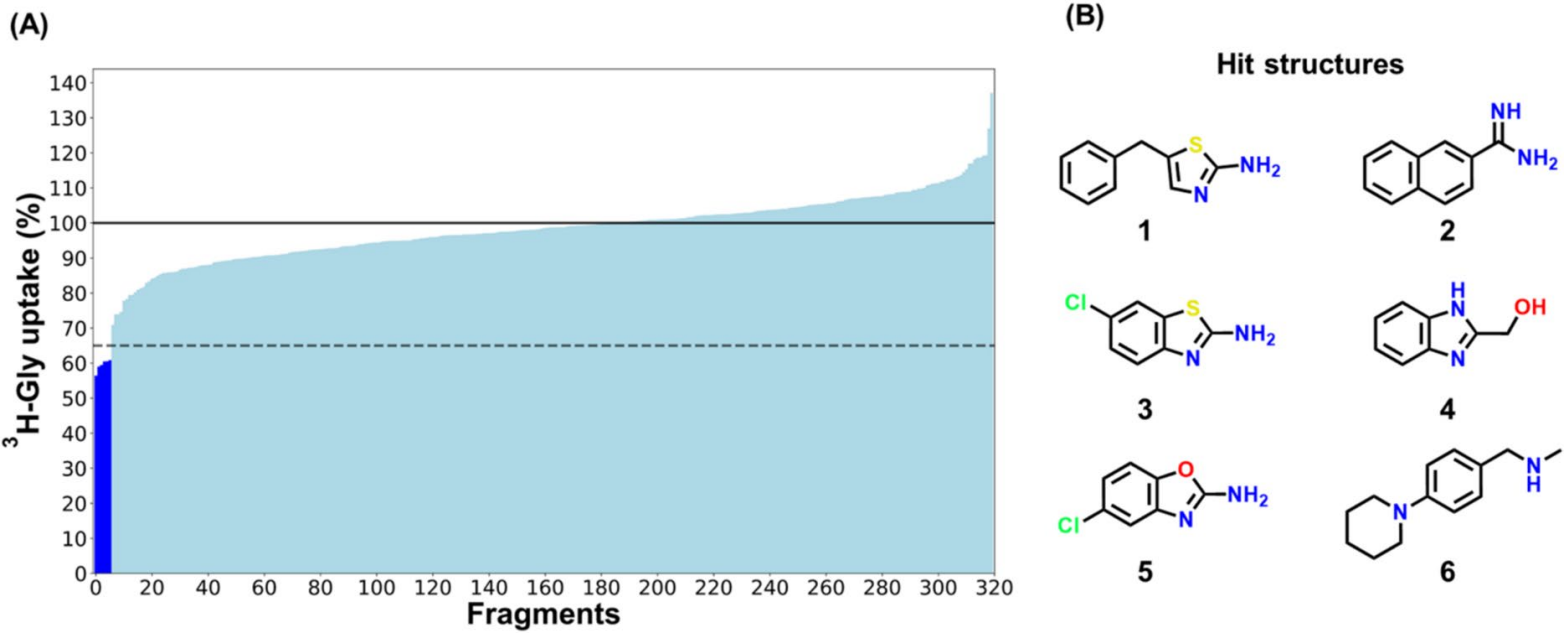
Screening of the Essential Fragment Library containing 320 fragment compounds for their inhibition of SNAT2 mediated ^3^H-Gly uptake in hyperosmotically treated PC-3 cells. (**A**) Waterfall plot showing the ranked effect of the library fragment compounds (0.5 mM) on a 5-min ^3^H-Gly (11.1 nM) uptake (*n* = 1, *N* = 1). Fragment compounds inhibiting uptake > 35% compared to the control were considered hits, as highlighted in the figure. All experiments used HBSS*. The cells were exposed to 0.5 µCi ⋅ mL^−1 3^H-Gly (11.1 nM) at 37 °C. (**B**) Chemical structures of the 6 identified hit fragment compounds

The Essential Fragment Library contains 320 fragment compounds with molecular weights in the range of 100–254 g/mol. These smaller molecules can serve as starting points for novel scaffolds for the further development of inhibitors. The library compounds were screened at 0.5 mM for their ability to inhibit SNAT2 in the ^3^H-Gly uptake assay, and the top 6 hits inhibited the uptake by > 35% (Fig. 2). Z’ factors were in the range of 0.69–0.80 for the screening study, showing excellent separation between the controls. Some common chemical features were seen in the 6 hits (Fig. 2B), like the 2-aminothiazole or 2-aminooxazole moiety either fused to a benzene ring or linked as a benzyl group as seen in **1**, **3**, and **5**. Interestingly, **5** is also known as the drug compound zoxazolamine, which was previously used as a muscle relaxant while also promoting the excretion of uric acid [20]. However, it was later withdrawn due to cases of hepatotoxicity [21].

**Fig. 3:**
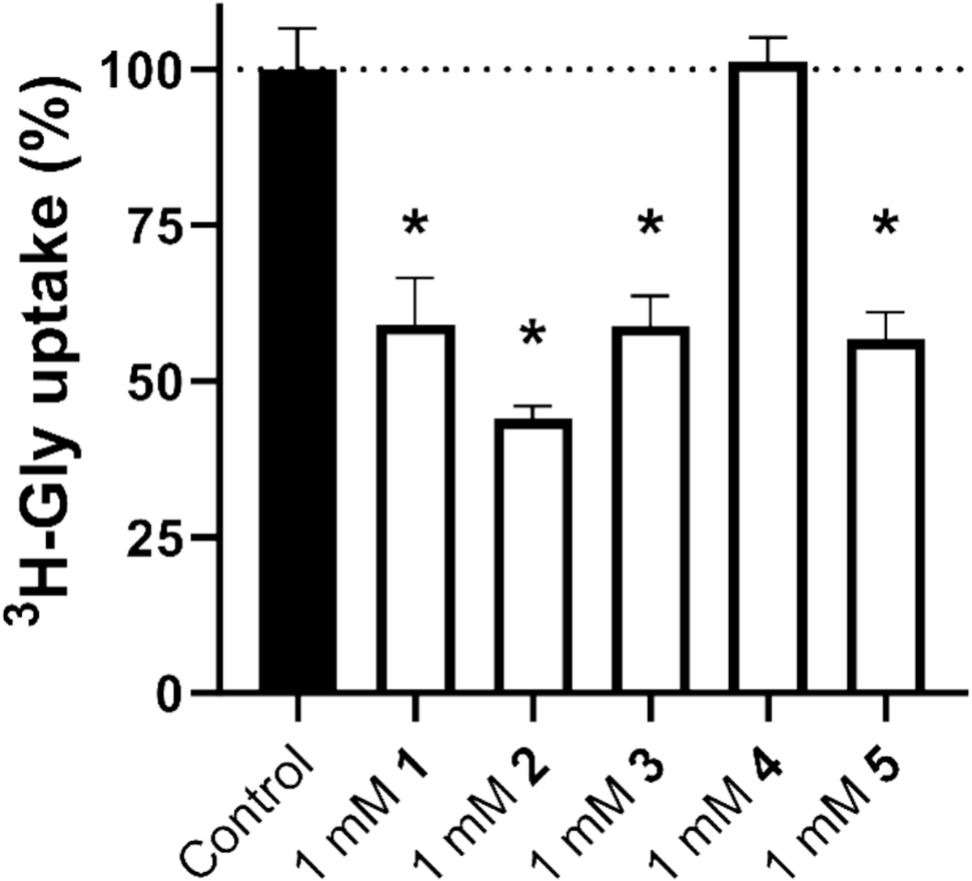
Normalized ^3^H-Gly uptake in hyperosmotically treated PC-3 cells (*n* = 3) in the absence (control, *N* = 18) or presence of 1 mM of the fragment compound hits (*N* = 9, except **2**, where *N* = 6). All experiments used HBSS*, pH 7.4. The cells were exposed to 0.5 µCi ⋅ mL^−1 3^H-Gly (11.1 nM) for 5 min at 37 °C. Values are reported as means ± SD, and statistically significant differences from the control identified in a one-way ANOVA followed by Dunnett's multiple comparisons test are highlighted (*, p < 0.05)

To validate that the observed hits inhibited SNAT2, **1–5** were bought as solid compounds and tested in biological replicates for their ability to inhibit ^3^H-Gly uptake at 1 mM (Fig. 3). All of the compounds appeared to significantly inhibit the uptake, except for **4**, which appeared to be a false positive from the screen. Compounds **1**,** 2**,** 3**, and** 5** thus appeared to be novel non-amino acid inhibitors of SNAT2. To help illustrate the structural uniqueness of the hit compounds compared to the rest of the fragment-based library, a hierarchal clustering based on a Tanimoto similarity coefficient matrix was employed and visualized as a heatmap (Supplementary Information, Figure S1). Interestingly, compounds **1**, and **2** seem to cluster together, despite limited Tanimoto similarity. Compounds **3** and** 5** also cluster together but appear to be surrounded by a greater deal of similarity. To further extract similar compounds, the top five compounds with the largest Tanimoto coefficients to each of the four hits are highlighted in Figure S2 (Supplementary Information). Interestingly, many compounds seem to share a similar scaffold as **3**, with different abilities to affect SNAT2-mediated ^3^H-Gly uptake. While these observations are only based on a single repetition, a more detailed exploration of the structure–activity relationship (SAR) was sought.

### Structure–Activity Relationship

Given the structural similarity of the hits, analogs that expanded on the hit scaffolds (primarily the 1,3-benzothiazole-2-amine scaffold) were studied to gain insight into the SAR. Their inhibition of SNAT2-mediated ^3^H-Gly uptake can be seen in Table 1. Focusing on the SAR of the 1,3-benzothiazole-2-amine scaffold, it is seen that the non-substituted scaffold cannot inhibit SNAT2 at 1 mM when comparing **3** to **7**. The same appears to be true for the 1,3-benzoxazole-2-amine scaffold, comparing **5** to **24**. Looking at the positioning of the chloro substituent in the benzothiazole scaffold, it is seen that only positions 6 and 7 (corresponding to compounds **3** and **8,** respectively) show inhibition, while 5-chloro-1,3-benzothiazol-2-amine (**9**) and 4-chloro-1,3-benzothiazol-2-amine (**10**) show no inhibition. Interestingly, the benzoxazole scaffold appears to be different, given that **5** shows inhibition, despite the chloro group being in the 5-position. As for which substituents that led to inhibition of SNAT2, hydrophobic groups like halogens (**3**, **5**, **11**), methyl (**12**), and ethyl (**14**) groups appear favorable, while hydrophilic groups like hydroxyl (**17**), amino (**20**), carboxyl (**21**), and nitro (**25**) groups appear unfavorable. As for the halogens, the smaller-sized chloro group (**3**) appears to be better than the bigger bromo group (**11**), something that was also indicated by the initial screen (Supplementary Information, Figure S2). The trifluoromethoxy group seen in **18**, also known as the drug compound riluzole used to treat amyotrophic lateral sclerosis (ALS) [22], initially appeared to be favorable for SNAT2 inhibition, but this compound also showed a similar decrease in cellular ATP levels as seen in the CellTiter-Glo assay, linking the observed inhibition to a possible cytotoxic effect. Other notable takeaways are that the ring closure of **1** seen in **23** appears to have a negative impact on its inhibitory activity and that the naphthalene amidine (**2**) is favorable to that of the phenyl amidine (**28**).

**Table 1:**
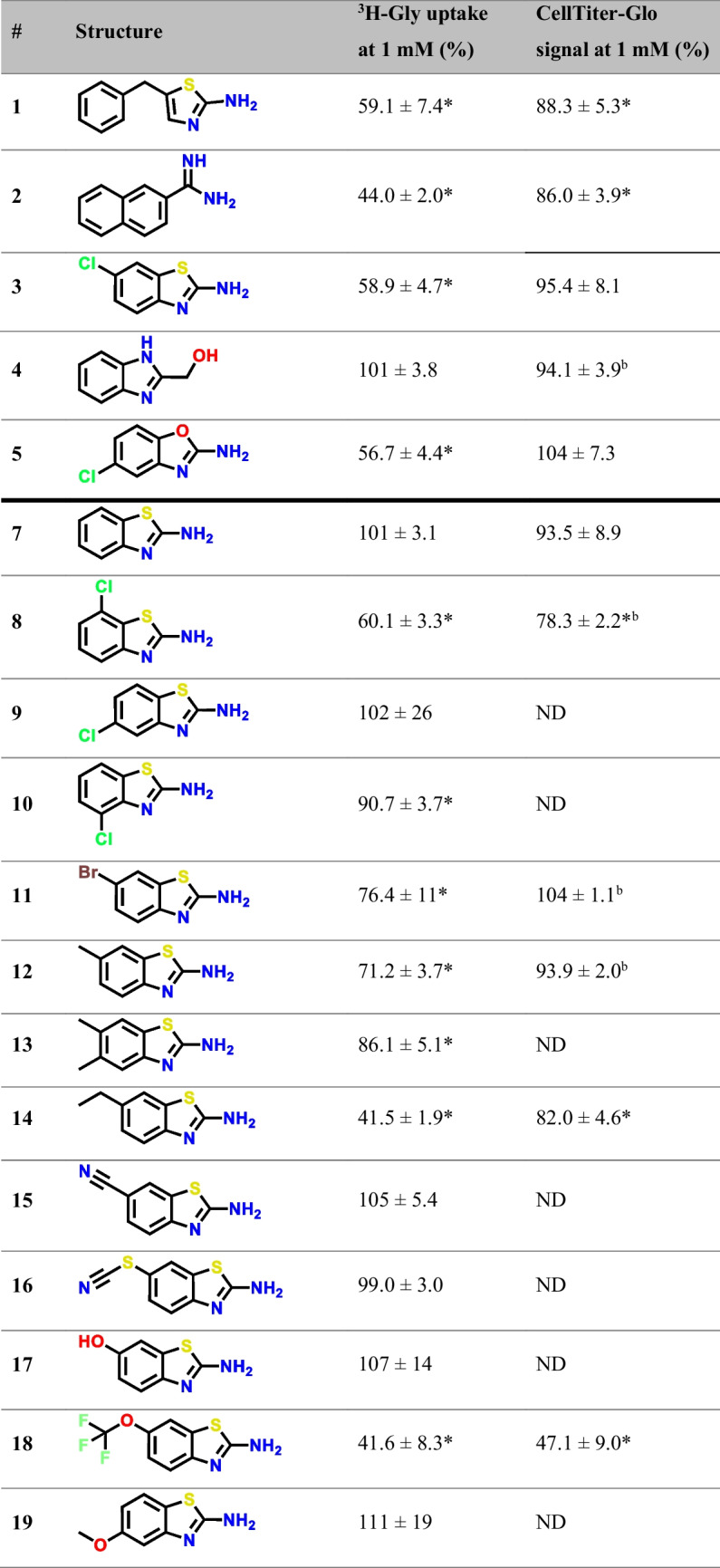

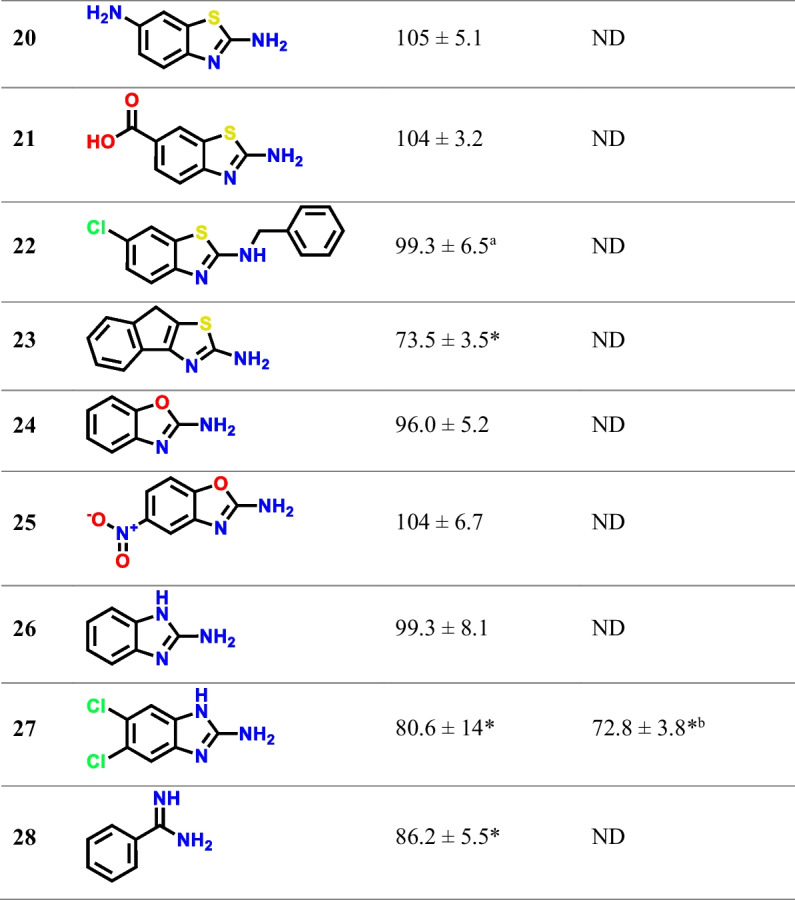
Inhibition of ^3^H-Gly uptake (*n* = 3, *N* = 6–9) and effect on CellTiter-Glo viability assay (*n* = 3–4, *N* = 9–13) in hyperosmotically treated PC-3 cells by the identified fragment compound hits and analogs at 1 mM concentrations. Values are reported as means ± SD, and statistically significant differences from the control identified in a one-way ANOVA followed by Dunnett's multiple comparisons test are highlighted (*, p < 0.05)

^a^Tested at 0.5 mM

^b^*n* = 1, *N* = 3–4

**Fig. 4:**
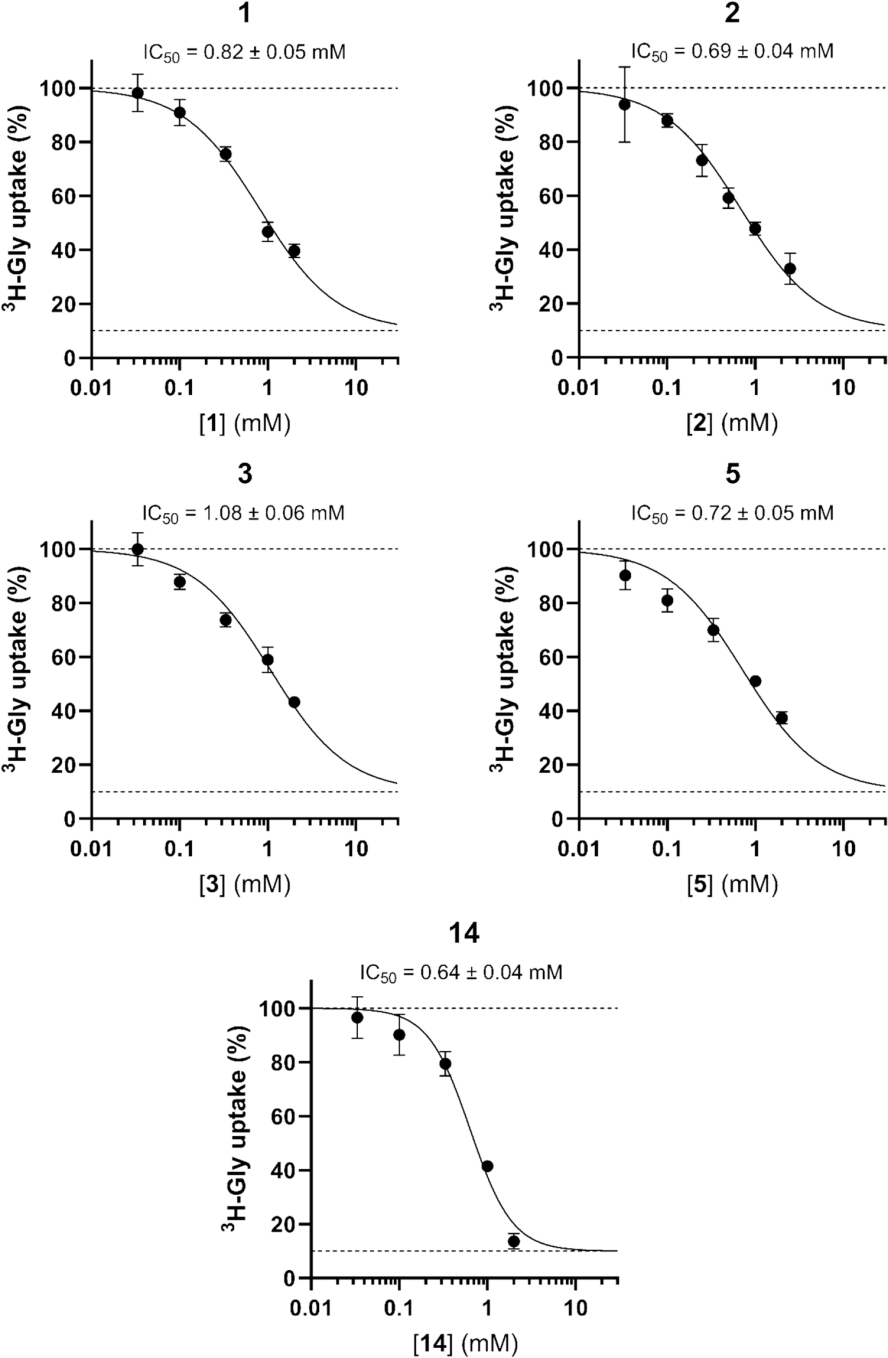
Concentration-dependent inhibition of ^3^H-Gly uptake in hyperosmotically treated PC-3 cells by **1**, **2**, **3**, **5**, and **14** (*n* = 3, *N* = 6–9). All experiments used HBSS*. The cells were exposed to 0.5 µCi ⋅ mL^−1 3^H-Gly (11.1 nM) for 5 min at 37 °C. Values are reported as means ± SD. The points were fitted to **Eq. **(2) to determine the IC_50_ value (± SE), with the Top and Bottom parameters being constrained to 100% and 10%, respectively. All curves were fitted to a standard Hill slope of −1, except **14,** which was fitted to a Hill slope of −1.8

The compounds **1**, **2**, **3**, **5**, and **14** appeared to be the most promising SNAT2 inhibitors and were thus subjected to concentration-dependent studies to determine their IC_50_ values (Fig. 4). Because of solubility limitations, it was difficult to attain high enough concentrations to profile the bottom of the IC_50_-curve, which is why the bottom was constrained to the value achieved by the positive control (20 mM L-Gln). For **1**, **2**, **3**, and **5,** the IC_50_ curves were comparable with IC_50_s in the range of 0.69–1.08 mM. **14** appeared to be the most potent inhibitor based on its IC_50_ value but also showed a noticeably steeper decrease in ^3^H-Gly uptake with increased concentrations. This indicated possible cytotoxic effects of **14,** and at 2 mM, **14** was shown to decrease the CellTiter-Glo signal to 36.6% of the control (Supplementary Information, Figure S3A). Interestingly, all of the compounds (except **5**) tested for their concentration dependence showed a marked decrease in the CellTiter-Glo signal at their highest tested concentration (Supplementary Information, Figure S3A). However, when the cells were allowed to recover in cell culture media for 30 min after exposure to the compounds, their ATP levels seemed to restore and were comparable with the control, except for 2 mM **18** (Supplementary Information, Figure S3B). Furthermore, when tested in the XTT viability assay, only 2 mM **18** seemed to drastically impact the cell viability, while a slight effect was seen for 2 mM **14** (Supplementary Information, Figure S4). Thus, it appears that the effect seen in the CellTiter-Glo assay for the majority of these compounds is more of a transient decrease in the ATP levels. Looking at the compound's effect at 1 mM on ^3^H-Leu uptake, which we have used to study LAT1 activity in PC-3 cells, none of the compounds appeared to signifcantly inhbit the uptake (Supplementary Information, Figure S5). Thus, the observed inhibition appears to be selective to SNAT2 compared to LAT1. If a cytotoxic effect was the reason for the observed inhibition of ^3^H-Gly uptake, then the same would be expected in the ^3^H-Leu uptake. Especially since the isoosmotically treated PC-3 cells used for the ^3^H-Leu uptake assay appear to be more susceptible to a reduction in the CellTiter-Glo signal compared to the hyperosmotically treated PC-3 cells used for the ^3^H-Gly uptake assay (Supplementary Information, Figure S6). It thus seems that cytotoxicity confounding the uptake results is mostly a problem for high concentrations of **14** and **18**.

### FLIPR Membrane Potential (FMP) Assay

**Fig. 5:**
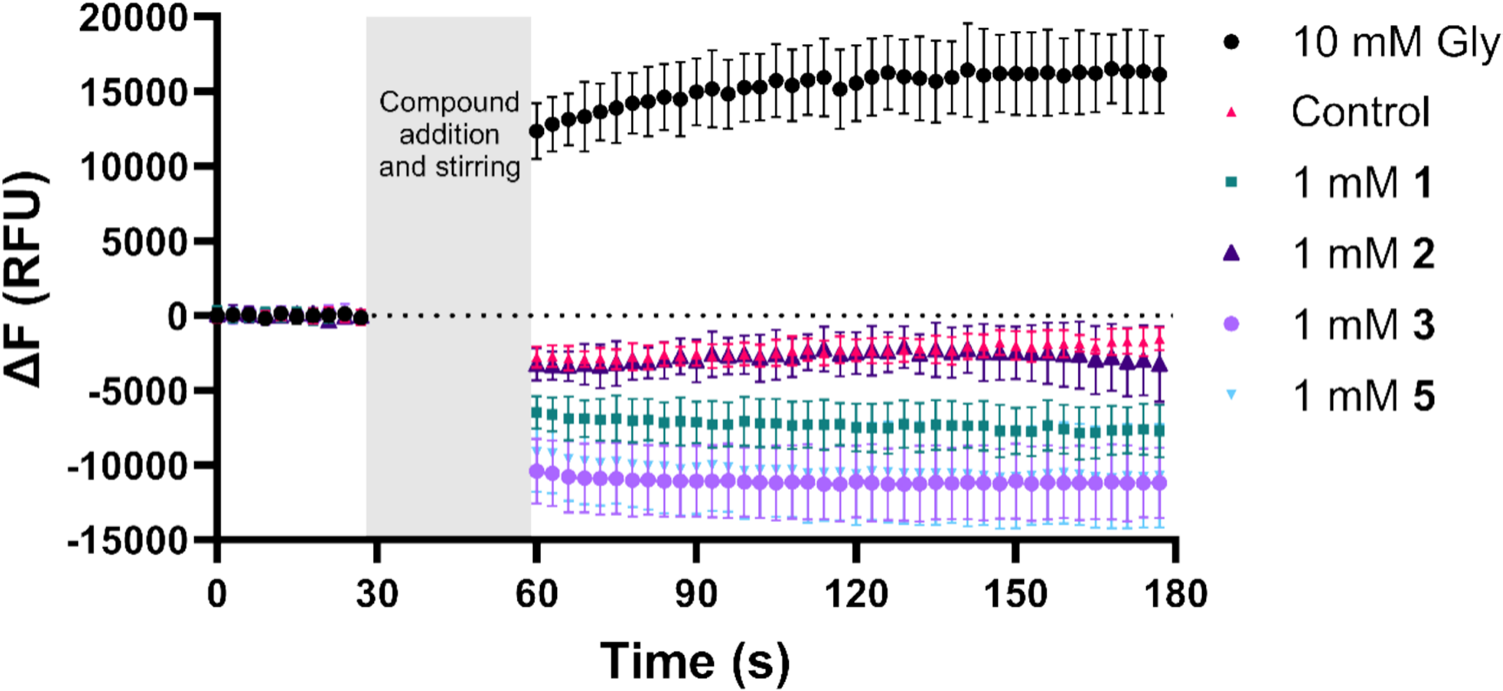
Time-response curve showing the change in fluorescence from the baseline (ΔF) in the FLIPR membrane potential (FMP) assay in hyperosmotically treated PC-3 cells (*n* = 3, *N* = 7–9). All experiments used HBSS* and an FMP probe concentration of 0.55 mg ⋅ mL^−1^. Fluorescence measurements (Ex. 530 nm, Em. 565 nm) were done 30 s before compound addition to establish the baseline, and the following gap reflects the time used to add the compound solutions. After compound addition, the fluorescence was measured for 120 s. Values are represented as means ± SD

To study if the identified fragment compounds were transported by SNAT2, the FLIPR membrane potential (FMP) assay was used. We have previously shown that SNAT2 substrates increased the fluorescent signal in the FMP assay in hyperosmotically treated PC-3 cells due to the depolarization caused by the inward Na^+^ current carried by SNAT2 [12]. Fig. 5 shows the FMP traces following the addition of the SNAT2 substrate Gly or the compounds **1**, **2**, **3**, and **5**. It is seen that the addition of Gly increases the fluorescent signal from the baseline as expected by a substrate. Interestingly, **1**, **3**, and **5** appear to decrease the signal, suggesting that they lead to membrane hyperpolarization. These structures are similar to riluzole (**18**), which is known to interact with various ion channels [23–25]. Interactions with ion channels could be the cause of the observed hyperpolarization of the cells, and riluzole has been shown to cause hyperpolarization of PC-3 cells through activation of the potassium channel TREK-1 [26]. Furthermore, both riluzole and zoxazolamine (**5**) have been shown to activate the SK2 potassium channel, which would increase the potassium conductance and hyperpolarize the membrane [23, 27]. These interactions with ion channels can perhaps also explain the observed fall in ATP levels, given that the Na^+^/K^+^-ATPase might consume an increased amount of ATP to restore the ion gradients. On the other hand, the FMP trace for 1 mM **2**, which does not equally resemble riluzole structurally, closely follows the background control trace and, therefore, does not appear to affect the membrane polarization of the cells.

**Fig. 6:**
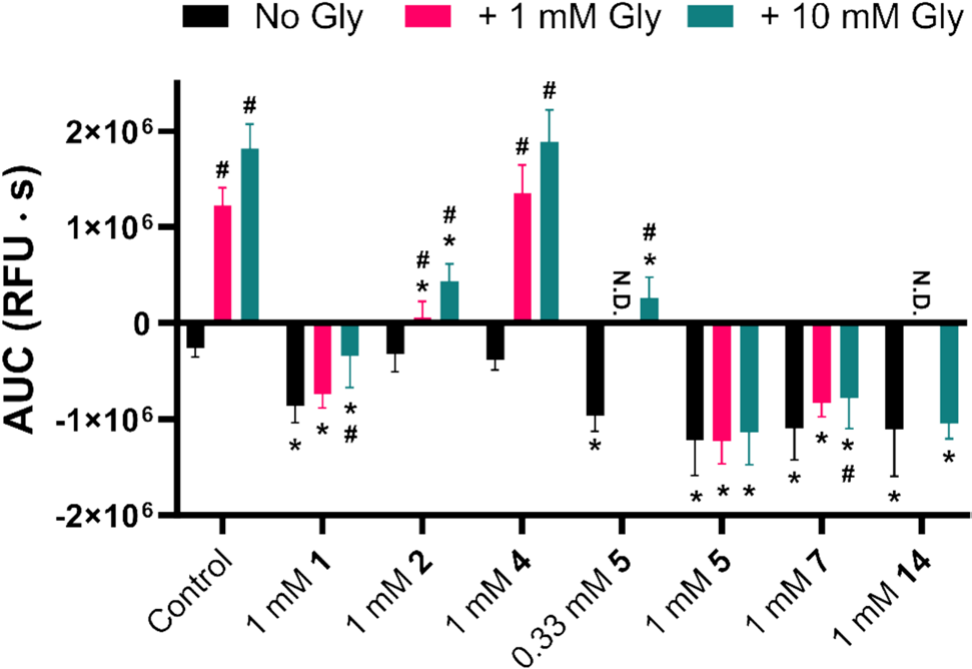
Area under the curve (AUC) of FMP curves of fragment compounds in hyperosmotically treated PC-3 cells in the absence or presence of 1 mM or 10 mM Gly (*n* = 3–5, *N* = 7–15). All experiments used HBSS*, and an FMP probe concentration of 0.55 mg ⋅ mL^−1^. After compound addition, the fluorescence (Ex. 530 nm, Em. 565 nm) was measured every 3 s for 120 s. The AUC reflects the change in fluorescence from the baseline (ΔF) after compound addition over time. Values are represented as means ± SD. Statistically significant differences (p < 0.05) revealed by two-way ANOVA followed by Šídák's multiple comparisons test are indicated with * when comparing to the control (in the same Gly condition) and with **#** when comparing to “No Gly” (in the same compound condition). N.D., not determined

To further investigate the dynamics of the fragment compounds in the FMP assay, they were studied together with the SNAT2 substrate Gly. This was done for two reasons. One was to see if the observed decrease in the FMP signal by the fragment compounds could be reversed by the addition of Gly. The second was to see if the Gly-induced FMP signal could be inhibited by the fragment compounds, as seen in the uptake studies. Fig. 6 shows the AUCs of the FMP curves of both SNAT2 uptake inhibiting (**1**, **2**, **5**, and **14**) and non-inhibiting (**4** and **7**) fragment compounds in the absence or presence of Gly. The apparent hyperpolarization seen by **1** appears to be moderately reversed by the addition of high concentrations of Gly. The same seems true for 0.33 mM **5**, but not in the case of 1 mM **5**. 1 mM **7** also appears to cause a hyperpolarization that is only slightly reversed by the addition of 10 mM Gly. However, **7** does not inhibit SNAT2 in uptake studies, indicating that ^3^H-Gly uptake inhibition is not linked to the hyperpolarizing effect. In fact, it has been shown that SNAT2 produces larger currents (indicating a higher transport rate) at lower membrane potentials [28, 29], again suggesting that a hyperpolarization would not be the cause of observed inhibition. **2** and **4** both seem to have no impact on membrane polarization on their own, but while **2** both inhibits ^3^H-Gly uptake and the FMP signal caused by Gly, **4** inhibits neither. **14** decreases the FMP signal, which is not reversed by the addition of 10 mM Gly. Because of the apparent hyperpolarization caused by **1**, **3**, **5**, and **14**, which is likely caused by interactions with targets other than SNAT2, we cannot resolve if they are translocated by SNAT2 or not. However, as **2** did not affect the membrane potential and showed inhibition in both the uptake and FMP assays, it appears to be a non-translocated inhibitor of SNAT2.

### Mechanism of Inhibition

**Fig. 7:**
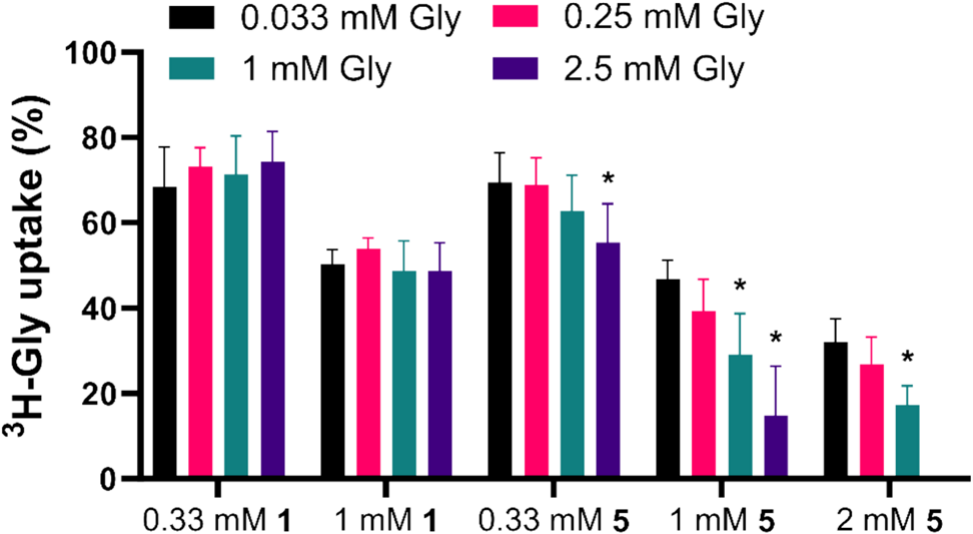
Normalized ^3^H-Gly uptake in hyperosmotically treated PC-3 cells in the presence of **1** or **5** at increasing concentration of cold Gly (*n* = 3, *N* = 6). All experiments used HBSS*. The cells were exposed to 0.5 µCi ⋅ mL^−1 3^H-Gly (11.1 nM) for 5 min at 37 °C. The uptake values were normalized to a control uptake with the same substrate concentration. Values are reported as means ± SD, and statistically significant differences from the uptake at 0.033 mM Gly identified in a two-way ANOVA followed by Dunnett's multiple comparisons test are highlighted (*, p < 0.05)

To gain insight into the mechanism of inhibition by the fragment compounds **1** and **5**, their inhibition of ^3^H-Gly uptake was studied at increasing concentrations of cold Gly. Looking at the uptake as % of the control, it is seen that the inhibition caused by **1** does not seem to be affected by differences in substrate concentration, suggesting a non-competitive type of inhibition (Fig. 7). On the other hand, **5** appears to show better inhibition of ^3^H-Gly uptake when the substrate concentration is increased, which is indicative of un-competitive inhibition. To confirm that these observations fit the different inhibition types, O-benzyl-L-serine was also studied, which was previously shown to be a substrate of SNAT2 [12] and thus should inhibit the uptake competitively. Here, the increase in substrate concentration decreased the observed inhibition by O-benzyl-L-serine as expected of a competitive inhibitor (Supplementary Information, Figure S7). Interestingly, **2** appeared to follow the same pattern, indicating that it also competed with the substrate to bind SNAT2 (Supplementary Information, Figure S7).

**Fig. 8:**
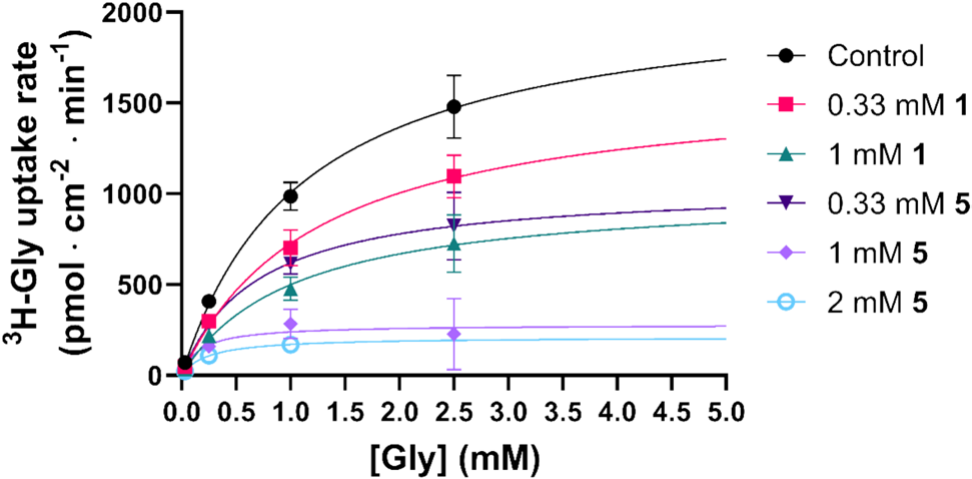
Concentration-dependent ^3^H-Gly uptake in hyperosmotically treated PC-3 cells in the absence or presence of different concentrations of **1** or **5** (*n* = 3, *N* = 6). All experiments used HBSS*. The cells were exposed to 0.5 µCi ⋅ mL^−1 3^H-Gly (11.1 nM) for 5 min at 37 °C. Values are reported as means ± SD, and each dataset was fitted to **Eq. **(3)

Plotting the observed uptakes as Michaelis–Menten plots (Fig. 8) allows for the determination of the V_max_ and K_m_ values and the comparison of how the fragment compounds affect these kinetic parameters (Table 2). It is seen that both **1** and **5** are able to lower the V_max_, with 1 mM **1** leading to a twofold reduction, while 1 mM **5** reduces the V_max_ almost tenfold. **1** does not seem to have a significant effect on the K_m_ value, whereas **5** leads to a significant lowering of the K_m_. This respectively fits the picture of a non-competitive inhibitor and an un-competitive inhibitor. However, a more nuanced view would be to look at this as different kinds of mixed inhibition, where the inhibitors are able to bind both the free transporter and the transporter-substrate complex. In the case of **1,** the affinities for both situations appear to be equal, while **5** appears to have a preference for the substrate-bound situation. In any case, it suggests that these fragment compounds are allosteric inhibitors of SNAT2, given that binding to the substrate binding site would result in competitive inhibition.

**Table 2:** Michaelis–Menten kinetic parameters of ^3^H-Gly uptake in hyperosmotically treated PC-3 cells in the absence or presence of different concentrations of **1** or **5** (*n* = 3, *N* = 6). Values are reported as means ± SE, and statistically significant differences from the control identified in a one-way ANOVA followed by Dunnett's multiple comparisons test are highlighted (*, p < 0.05)

	V_max_ (pmol ⋅ cm^−2^ ⋅ min^−1^)	K_m_ (mM)
Control	2130 ± 97.9	1.12 ± 0.12
0.33 mM **1**	1628 ± 123.3*	1.25 ± 0.21
1 mM **1**	1013 ± 113.4*	1.03 ± 0.28
0.33 mM **5**	1049 ± 89.0*	0.69 ± 0.17
1 mM **5**	279 ± 46.2*	0.17 ± 0.13*
2 mM **5**	211 ± 22.5*	0.24 ± 0.08*

In an earlier study, Erickson identified a high-affinity glutamine transporting system in hippocampal neurons that is reminiscent of the system A SNAT transporters (SNAT1, SNAT2, SNAT4) [30]. Both have an affinity for 2-(methylamino)isobutyrate (MeAIB) and similar substrate profiles, but the orphan system has better affinities for its substrates compared to that of SNAT1 and SNAT2 [30]. Interestingly, this high-affinity system is also inhibited by riluzole and related structures [30, 31]. In fact, there is some overlap in the structure–activity relationship seen here for SNAT2 and what has been described for the high-affinity SNAT-like system. For instance, in both cases, the non-substituted 1,3-benzothiazole-2-amine scaffold shows limited inhibition, substitution at the 6-position appears to be favored, and amine or carboxyl groups at the 6-position show weak inhibition, while a chloro group (as seen in **3**) shows great inhibition [31]. Although these riluzole-related compounds show better inhibitory potencies for the high-affinity system (> 100-fold difference), it is quite noteworthy that this inhibitor scaffold has been discovered for two similar transport systems through completely different and independent methods. It could suggest that the high-affinity system is an orphan SLC38/SNAT member and that a somewhat conserved allosteric site exists where these inhibitors might bind. While Kyllo et al. proposes that inhibitors enact their inhibition through an indirect mechanism involving a Ca^2+^ dependent cycling of an intracellular store of transporters [31], we suggest that the inhibitors bind allosterically to the transporter given the short time 5-min exposure and because the inhibition has been observed in different cell lines (neurons versus prostate cancer cells). In any case, these 1,3-benzothiazole-2-amines and 1,3-benzoxazole-2-amines appear to be a novel class of inhibitors that can alter the activity of SNAT-like transporters. Still, these compounds are of low potency when it comes to SNAT2 inhibition, but present a great opportunity, as these scaffolds, when further optimized by medicinal chemistry approaches, can become valuable chemical tools to study SNAT2 and related transporters.

## Conclusion

By screening a fragment-based library, we discovered a novel set of non-amino acid inhibitors of SNAT2, primarily based on 1,3-benzothiazole-2-amines and 1,3-benzoxazole-2-amine scaffolds. These inhibitors inhibited SNAT2-mediated ^3^H-Gly uptake with IC_50_ values in the range of 0.64–1.08 mM. Furthermore, they appeared to work through a mix of non-competitive and un-competitive inhibitory mechanisms and thus seem to be promising candidates for the development of drugs or chemical tools that allosterically target SNAT2.

## Supplementary Information

Below is the link to the electronic supplementary material.

Supplementary file1 (DOCX 1.47 MB) The Supplementary Information contains more detailed information on the compounds and vendors, Tanimoto similarities, CellTiter-Glo viability results, XTT viability results, ^3^H-Leu uptake results, and inhibitory mechanism studies.

## Data Availability

The datasets generated during and/or analyzed during the current study are available from the corresponding author on reasonable request.
