# Supplementary material for: Fragment-Based Screening Identifies Novel Non-Amino Acid Inhibitors of the Sodium-Coupled Neutral Amino Acid Transporter SNAT2
Source: Pharm Res. 2025 Aug 8;42(8):1285–97. doi: 10.1007/s11095-025-03902-7 (PMC12405023; doi:10.1007/s11095-025-03902-7)
Supplement: Supplementary file 1 — Supplementary file1 (DOCX 1.47 MB) The Supplementary Information contains more detailed information on the compounds and vendors, Tanimoto similarities, CellTiter-Glo viability results, XTT viability results, 3H-Leu uptake results, and inhibitory mechanism studies. [file 11095_2025_3902_MOESM1_ESM.docx]

Supplementary Information

Pharmaceutical Research

**Fragment-Based Screening Identifies Novel Non-Amino Acid Inhibitors of the Sodium-Coupled Neutral Amino Acid Transporter SNAT2**

Sebastian Jakobsen, Carsten Uhd Nielsen*

Department of Physics, Chemistry and Pharmacy, University of Southern Denmark, Campusvej 55, DK-5230 Odense M, Denmark.

*: Correspondence: Carsten Uhd Nielsen, Department of Physics, Chemistry and Pharmacy, University of Southern Denmark, Campusvej 55, DK-5230 Odense M, Denmark. Phone: +45 6550 9427, e-mail: cun@sdu.dk

# Compounds and Vendors

**Table S1** List of compounds **1**-**28** with the vendors they are acquired through and their purity.

| **#** | **Name** | **Vendor** | **Purity, %** |
| --- | --- | --- | --- |
| **1** | 5-Benzylthiazol-2-amine | BLDPharm | 98 |
| **2** | Naphthalene-2-carboximidamide | Enamine | 95 |
| **3** | 6-Chlorobenzothiazol-2-ylamine | BLDPharm | 98 |
| **4** | (1H-Benzoimidazol-2-yl)methanol | BLDPharm | 97 |
| **5** | 5-Chlorobenzo[d]oxazol-2-amine | BLDPharm | 97 |
| **7** | 1,3-benzothiazol-2-amine | Enamine | 95 |
| **8** | 7-chloro-1,3-benzothiazol-2-amine | Enamine | 95 |
| **9** | 2-Amino-5-chlorobenzothiazole | BLDPharm | 97 |
| **10** | 4-chloro-1,3-benzothiazol-2-amine | Enamine | 95 |
| **11** | 6-bromo-1,3-benzothiazol-2-amine | BLDPharm | 98 |
| **12** | 6-methyl-1,3-benzothiazol-2-amine | Enamine | 95 |
| **13** | 5,6-dimethyl-1,3-benzothiazol-2-amine | Enamine | 95 |
| **14** | 6-ethyl-1,3-benzothiazol-2-amine | Enamine | 95 |
| **15** | 2-amino-1,3-benzothiazole-6-carbonitrile | Enamine | 95 |
| **16** | [(2-amino-1,3-benzothiazol-6-yl)sulfanyl]formonitrile | Enamine | 95 |
| **17** | 2-Aminobenzothiazol-6-ol | BLDPharm | 97 |
| **18** | 6-(Trifluoromethoxy)benzo[d]thiazol-2-amine | BLDPharm | 97 |
| **19** | 5-Methoxybenzo[d]thiazol-2-amine | BLDPharm | 97 |
| **20** | 1,3-benzothiazole-2,6-diamine dihydrochloride | Enamine | 95 |
| **21** | 2-amino-1,3-benzothiazole-6-carboxylic acid | Enamine | 95 |
| **22** | N-benzyl-6-chloro-1,3-benzothiazol-2-amine | Enamine | 98 |
| **23** | 8H-indeno[1,2-d][1,3]thiazol-2-amine | Enamine | 95 |
| **24** | 1,3-benzoxazol-2-amine | Enamine | 95 |
| **25** | 5-Nitrobenzo[d]oxazol-2-amine | BLDPharm | 97 |
| **26** | 1H-Benzo[d]imidazol-2-amine | BLDPharm | 98 |
| **27** | 5,6-Dichloro-1H-benzo[d]imidazol-2-amine | BLDPharm | 96 |
| **28** | Benzimidamide hydrochloride | BLDPharm | 97 |


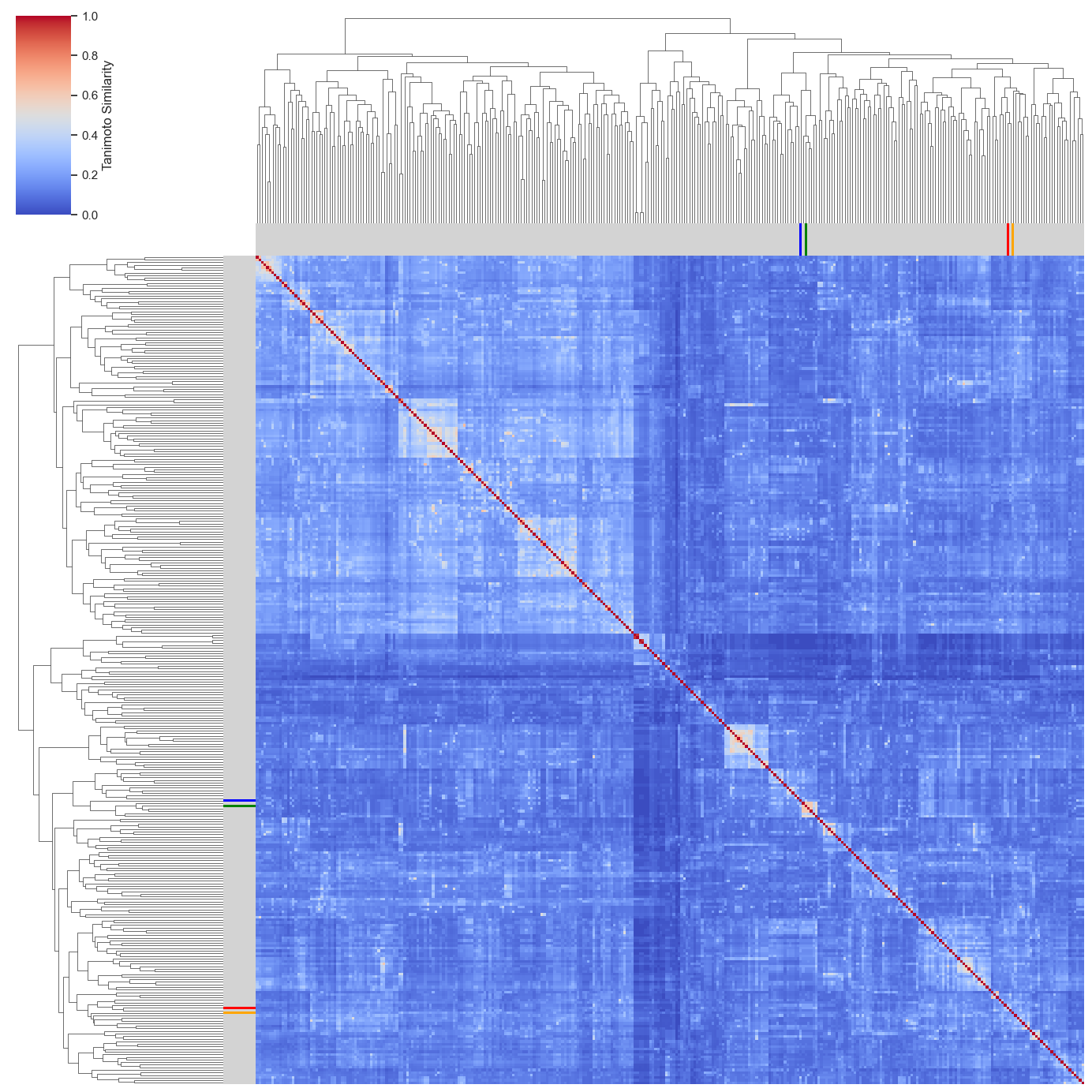
**Figure S1** Hierarchical clustering of the 320 fragment-sized compounds based on structural similarity. The heatmap displays pairwise Tanimoto similarity values (color scale from low [blue] to high [red]) calculated from 1024-bit Morgan fingerprints (*radius = 2*) generated using RDKit. Rows and columns represent individual compounds and are ordered by agglomerative hierarchical clustering using average linkage and a distance metric of *1 − Tanimoto similarity*. Dendrograms depict the resulting cluster hierarchy. The hit compounds **1**, **2**, **3**, and **5** are highlighted by the colors red, orange, green, and blue respectively.


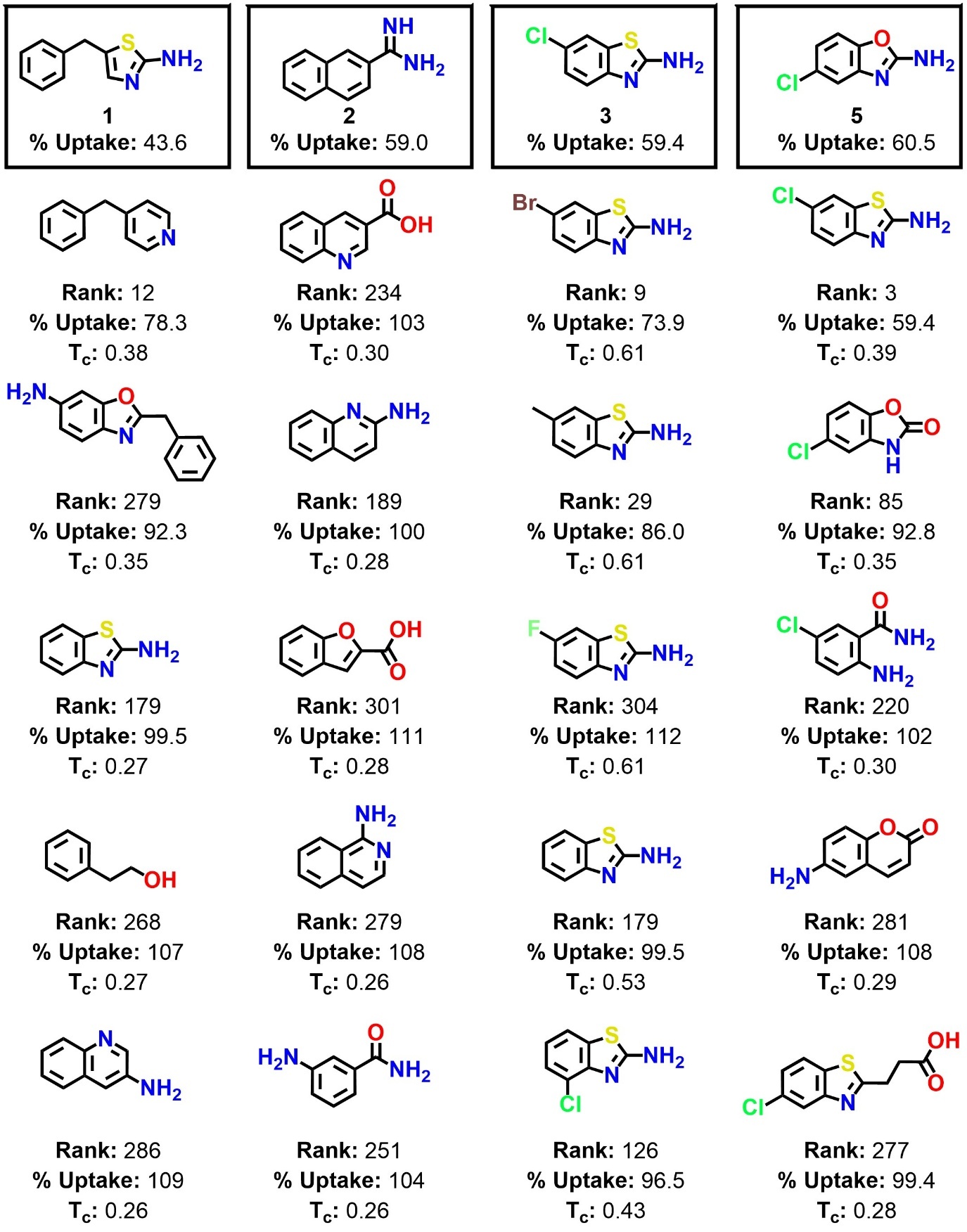


**Figure S2** Top five most similar compounds in the 320 fragment-sized compound library to the four confirmed hit compounds. Similarity was based on Tanimoto coefficients (T_c_) calculated from 1024-bit Morgan fingerprints (*radius = 2*) generated using RDKit. Each compound is annotated with its rank in the experimental screen, the observed ^3^H-Gly uptake (%) when screened at 0.5 mM, and the Tanimoto coefficient when compared to the hit compounds at the top.


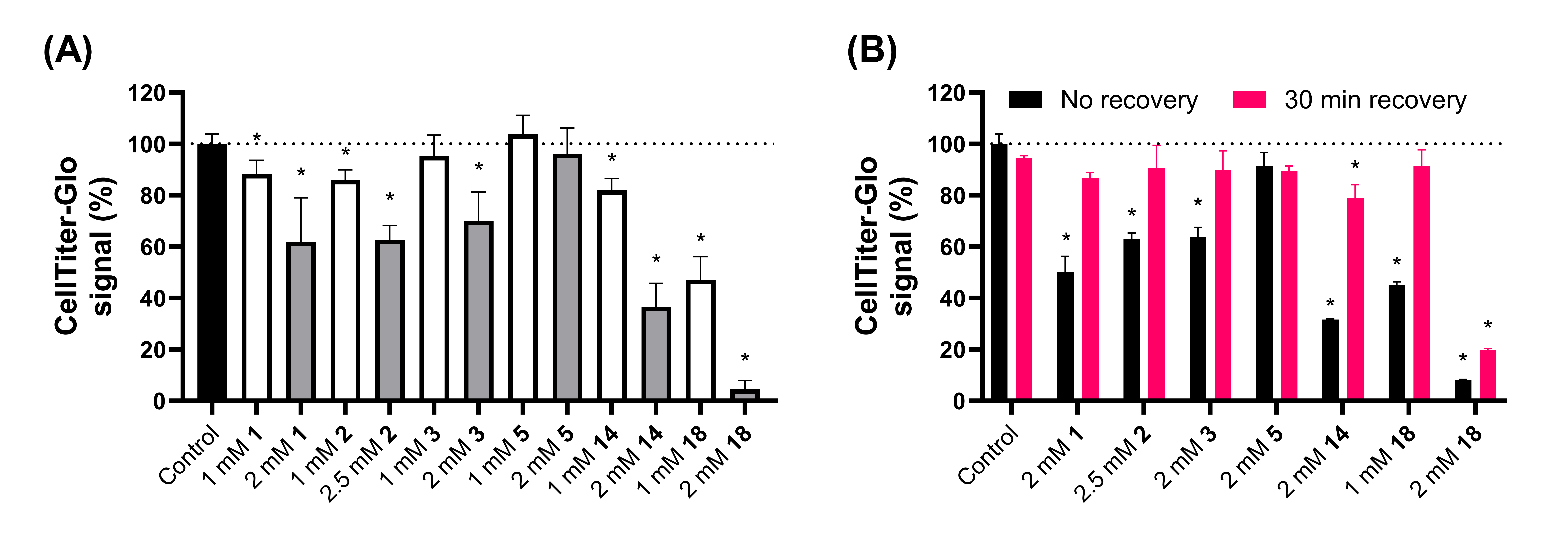


**Figure S3** Normalized CellTiter-Glo viability assay signals of hyperosmotically treated PC-3 cells. **(A)** CellTiter-Glo signal after 10 min incubation with fragment compounds (*n* = 3-4, *N* = 9-13 (Control *N* = 22)). **(B)** CellTiter-Glo signal after 10 min incubation with fragment compounds with or without a subsequent recovery period, where the cells are incubated in cell culture media for 30 minutes (*n* = 1, *N* = 3). All experiments used 10 mM HEPES buffer in HBSS, pH 7.4. Values are represented as means ± SD, and statistically significant differences from the control detected by one-way **(A)** or two-way **(B)** ANOVA followed by Dunnett's multiple comparisons test are shown (*: p < 0.05).


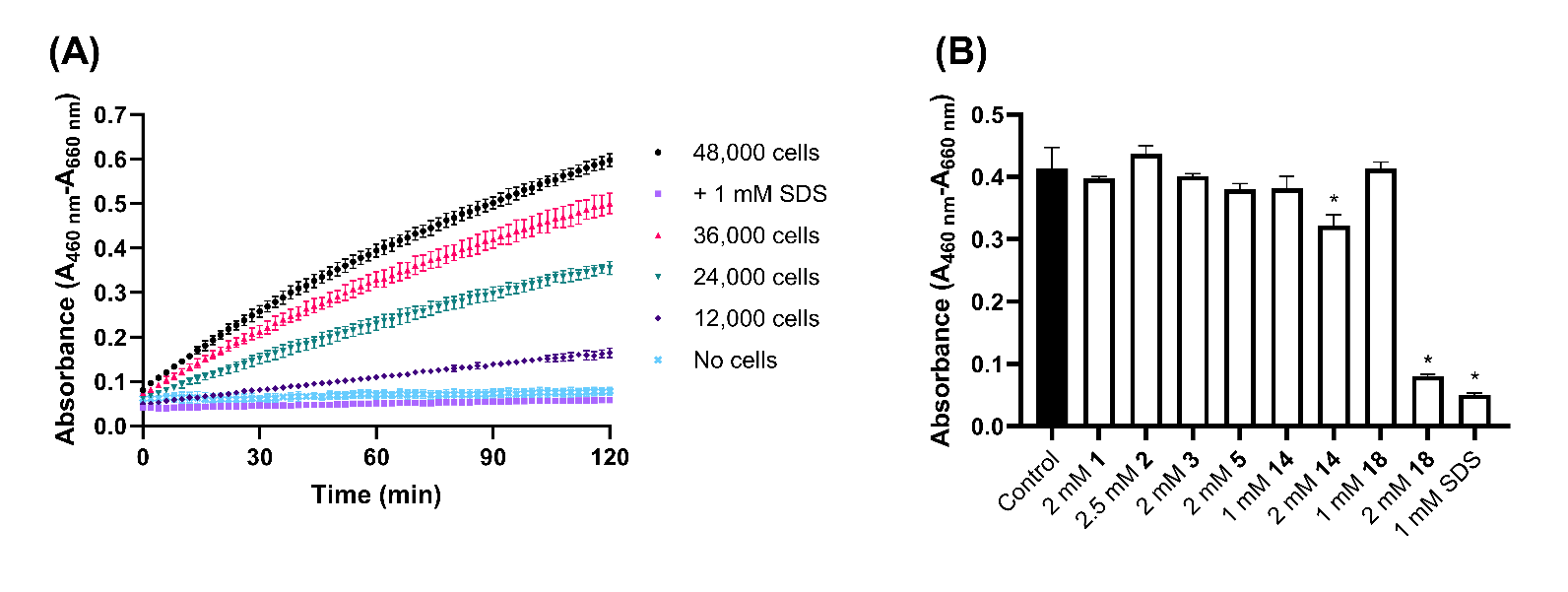


**Figure S4** XTT viability assay of hyperosmotically treated PC-3 cells (*n* = 1, *N* = 3-4). (**A**) Development in the XTT product absorbance over time at different cell seeding densities or after 10 min incubation with 1 mM SDS (48,000 cells seeded). (**B**) XTT product absorbance after 60 minutes for PC-3 cells treated with fragment compounds or 1 mM SDS for 10 minutes. PC-3 cells, seeded in clear 96-well plates (0.32 cm^2^) and treated with hyperosmotic media 24 hours before the experiment, were used for the viability assay. The XTT reagent was prepared on the day of the experiment by mixing XTT Na^+^ salt and phenazine methyl sulphate (PMS) for a final concentration of 0.9 mg ⋅ mL^−1^ XTT Na^+^ and 7.5 µg ⋅ mL^−1^ PMS in 10 mM HEPES buffer in HBSS, pH 7.4 (HBSS*). On the day of the experiment, the cells were preincubated in HBSS* for 15 minutes at 37°C and 220 rpm. The cells were then incubated with compounds for 10 minutes at 37°C and 220 rpm. The solutions were removed, and 60 µL of HBSS* was added to each well, along with 30 µL of the XTT reagent. The plate was placed in a CLARIOstar® Plus plate reader from BMG LABTECH (Ortenberg, Germany) at 37°C and was then shaken for 30 seconds at 200 rpm. The formation of the formazan product produced by viable cells was followed by measuring the absorbance at 460 nm every 2 minutes for a total duration of 120 minutes, using a reference wavelength of 660 nm. Values are represented as means ± SD. Statistically significant differences from the control detected by one-way ANOVA followed by Dunnett's multiple comparisons test are shown (*: p < 0.05).


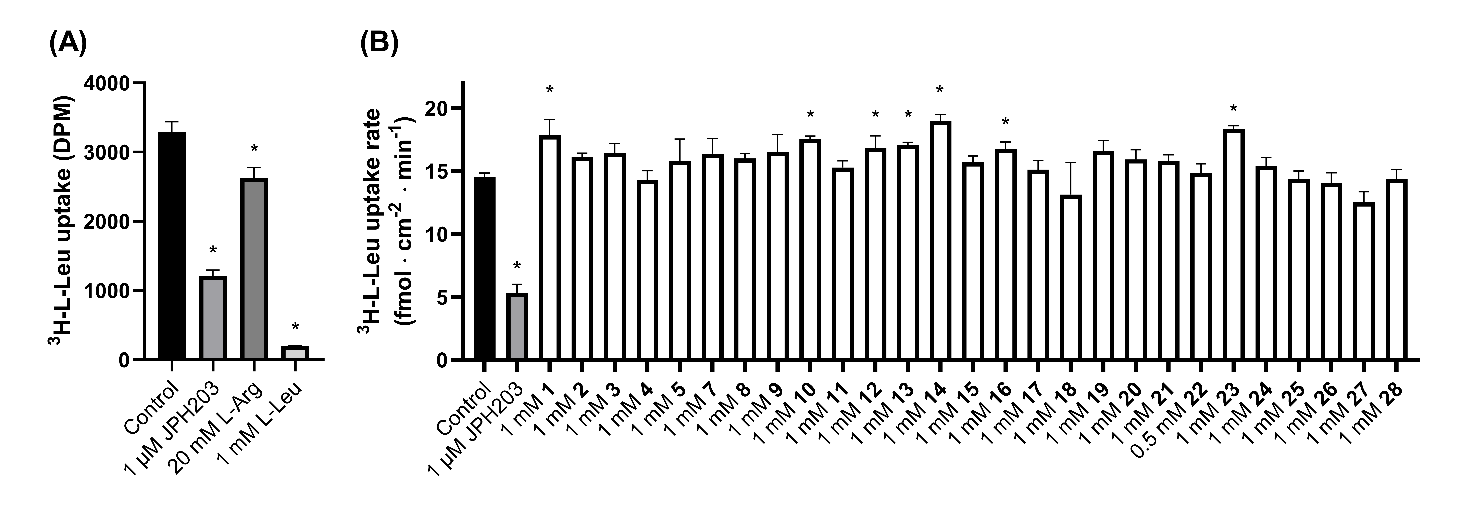


**Figure S5** ^3^H-L-Leu uptake in isoosmotically treated PC-3 cells. (**A**) ^3^H-L-Leu uptake (*n* = 1) in the absence (control, *N* = 8) or presence of the LAT1 selective inhibitor JPH203 (*N* = 8), the ATB^0,+^ and b^0,+^AT substrate L-Arg (*N* = 4), or 1 mM cold L-Leu (*N* = 4). (**B**) ^3^H-L-Leu uptake in the absence (control, *n* = 4) or presence of 1 µM JPH203 (*n* = 4) or compounds **1**-**28** (*n* = 3). All experiments used 10 mM HEPES buffer in HBSS, pH 7.4. The cells were exposed to 0.2 µCi ⋅ mL^−1^ ^3^H-L-Leu (2.75 nM) for 5 minutes at 37 °C. Values are reported as means ± SD, and statistically significant differences from the control identified in a one-way ANOVA followed by Dunnett's multiple comparisons test are highlighted (*, p < 0.05).


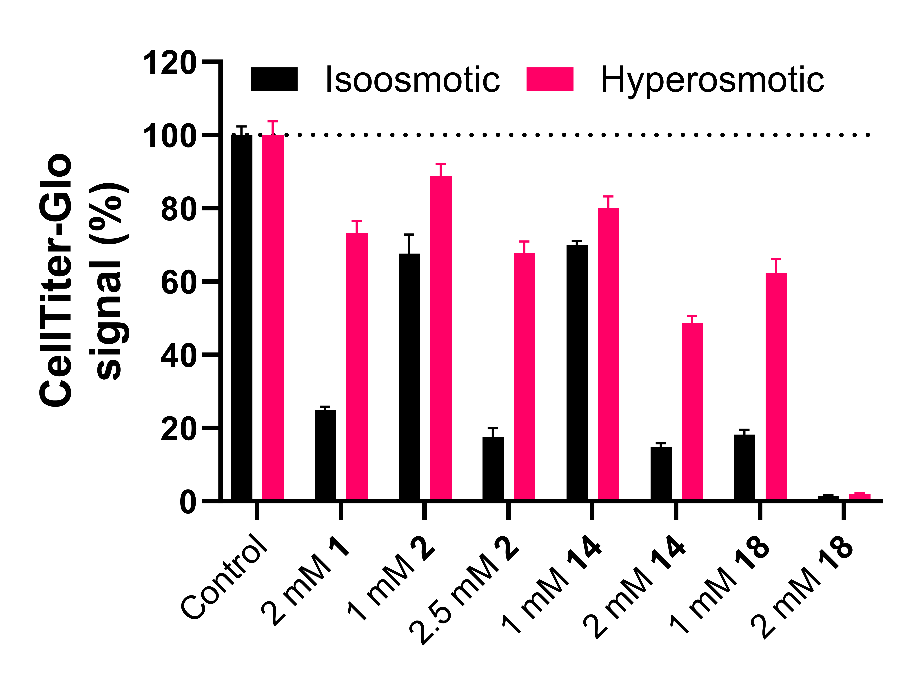


**Figure S6** Normalized CellTiter-Glo viability assay signals of isoosmotically or hyperosmotically treated PC-3 cells after 10 min incubation with fragment compounds (*n* = 1, *N* = 3-6). All experiments used 10 mM HEPES buffer in HBSS, pH 7.4. Values are represented as means ± SD. All datasets were significantly different from their controls as detected by one-way ANOVA followed by Dunnett's multiple comparisons test (p < 0.05).


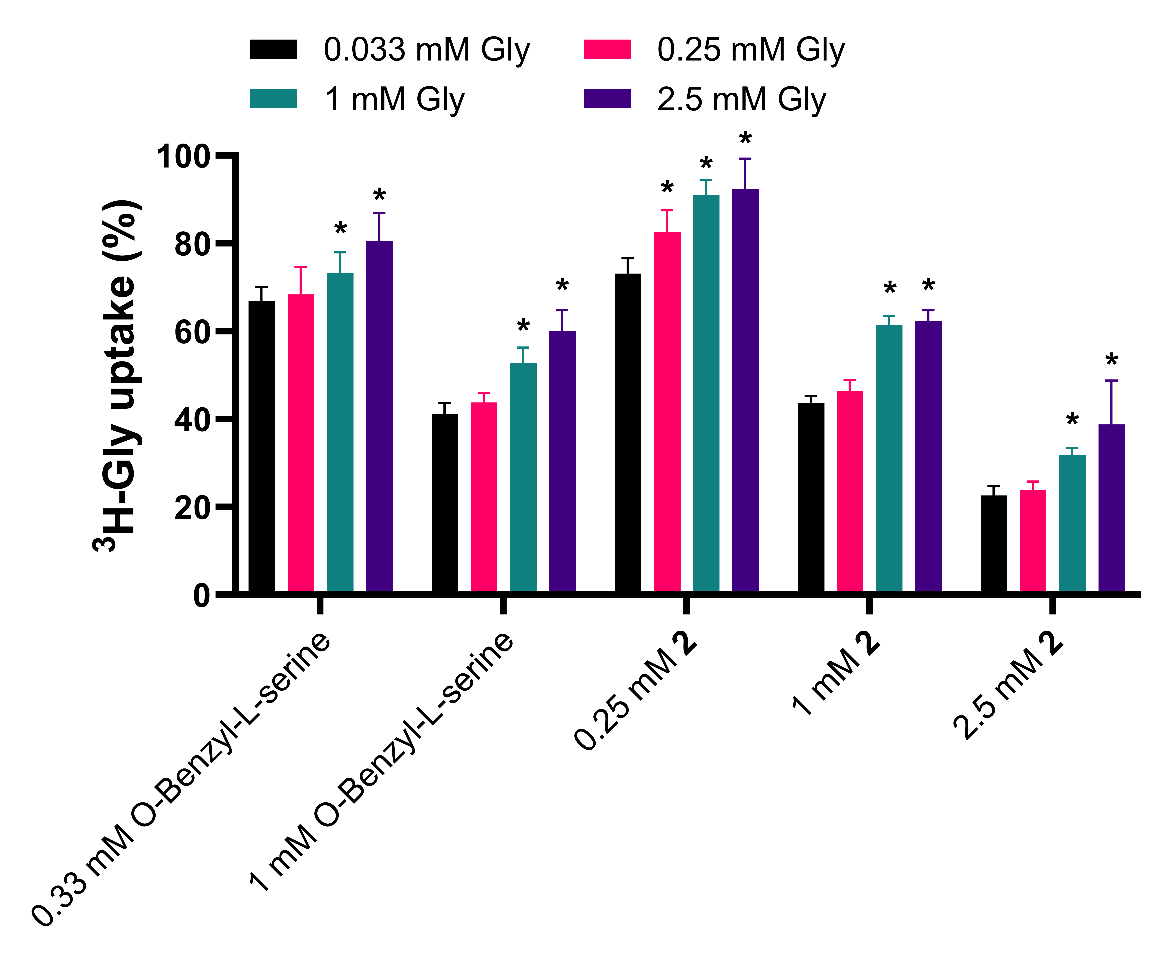


**Figure S7** Normalized ^3^H-Gly uptake in hyperosmotically treated PC-3 cells in the presence of O-benzyl-L-serine (*n* = 3, *N* = 6) or compound **2** (*n* = 1, *N* = 3) at increasing concentration of cold Gly. All experiments used 10 mM HEPES buffer in HBSS, pH 7.4. The cells were exposed to 0.5 µCi ⋅ mL^−1^ ^3^H-Gly (11.1 nM) for 5 minutes at 37 °C. Values are reported as means ± SD, and statistically significant differences from the uptake at 0.033 mM Gly identified in a one-way ANOVA followed by Dunnett's multiple comparisons test are highlighted (*, p < 0.05).
